# A Novel and Highly Effective Bayesian Sampling Algorithm Based on the Auxiliary Variables to Estimate the Testlet Effect Models

**DOI:** 10.3389/fpsyg.2021.509575

**Published:** 2021-08-11

**Authors:** Jing Lu, Jiwei Zhang, Zhaoyuan Zhang, Bao Xu, Jian Tao

**Affiliations:** ^1^Key Laboratory of Applied Statistics of MOE, School of Mathematics and Statistics, Northeast Normal University, Changchun, China; ^2^Key Lab of Statistical Modeling and Data Analysis of Yunnan Province, School of Mathematics and Statistics, Yunnan University, Kunming, China; ^3^Department of Statistics, School of Mathematics and Statistics, Northeast Normal University, Changchun, China; ^4^Institute of Mathematics, Jilin Normal University, Siping, China

**Keywords:** bayesian inference, deviance information criterion, logarithm of the pseudomarignal likelihood, item response theory, testlet effect models, slice-Gibbs sampling algorithm, Markov chain Monte Carlo

## Abstract

In this paper, a new two-parameter logistic testlet response theory model for dichotomous items is proposed by introducing testlet discrimination parameters to model the local dependence among items within a common testlet. In addition, a highly effective Bayesian sampling algorithm based on auxiliary variables is proposed to estimate the testlet effect models. The new algorithm not only avoids the Metropolis-Hastings algorithm boring adjustment the turning parameters to achieve an appropriate acceptance probability, but also overcomes the dependence of the Gibbs sampling algorithm on the conjugate prior distribution. Compared with the traditional Bayesian estimation methods, the advantages of the new algorithm are analyzed from the various types of prior distributions. Based on the Markov chain Monte Carlo (MCMC) output, two Bayesian model assessment methods are investigated concerning the goodness of fit between models. Finally, three simulation studies and an empirical example analysis are given to further illustrate the advantages of the new testlet effect model and Bayesian sampling algorithm.

## 1. Introduction

In education and psychological tests, a testlet is defined as that a bundle of items share a common stimulus (a reading comprehension passage or a figure) (Wainer and Kiely, 1987). For example, in a reading comprehension test, a series of questions may be based on a common reading passage. The advantages of the testlet design are not only to allow for more complicated and interrelated set of items, but also to improve the testing efficiency (Thissen et al., 1989). Namely, with several items embedded in a testlet, test takers need not waste a considerable amount of time and energy in processing a long passage just to answer a single item. Despite their appealing features, this testing format poses a threat to item analysis because items within a testlet often violate the local independence assumption of item response theory (IRT). The traditional item response analysis tends to overestimate the precision of person ability obtained from testlets, and overestimate test reliability\information, and yields biased estimation for item difficulty and discrimination parameters (Sireci et al., 1991; Yen, 1993; Wang and Wilson, 2005a; Wainer et al., 2007; Eckes, 2014; Eckes and Baghaei, 2015).

In the face of these problems, two methods have been proposed to cope with the local item dependence. One method is to estimate a unidimensional model but treat items within a testlet as a single polytomous item (Sireci et al., 1991; Yen, 1993; Wainer, 1995; Cook et al., 1999) and then apply polytomous item response models such as the generalized partial-credit model (Muraki, 1992), the graded response models (Samejima, 1969), or the nominal response model (Bock, 1972). This method is appropriate when the local dependence between items within a testlet is moderate and the test contains a large proportion of independent items (Wainer, 1995), but it becomes impractical as the number of possible response patterns increases geometrically with the number of items in a testlet and thus is not frequently used (Thissen et al., 1989). An alternative method is testlet effects can be taken into account by incorporating specific dimensions in addition to the general dimension into the IRT models. Two such multidimensional IRT models are often used by researchers. That is, the bi-factor models (Gibbons and Hedeker, 1992) and the random-effects testlet models (Bradlow et al., 1999; Wainer et al., 2007). However, Li et al. (2006), Rijmen (2010), and Min and He (2014) find that the random-effects testlet models can be used as a special case of the bi-factor models. It is obtained by constraining the loadings on the specific dimension to be proportional to the loading on the general dimension within each testlet. In practice, researchers prefer to use simple random-effects testlet models if the two models are available and the model fit is not too much damage. Next, we discuss the specific forms of some commonly used testlet effect models.

Several literatures on testlet structure modeling have been proposed to capture the local item dependence from different perspectives for the past two decades. Bradlow et al. (1999) and Wainer et al. (2000) extend the traditional IRT models including a random effect parameter to explain the interaction between testlets and persons. The probit link function of the above model is formulated as Φ[*a*_*j*_(θ_*i*_ − *b*_*j*_ + η_*id*(*j*)_)], where Φ is the normal cumulative distribution function, θ_*i*_ denotes the the ability for the *i*th examinee, *a*_*j*_ and *b*_*j*_, respectively denote the discrimination parameter and difficulty parameter for the *j*th item, and η_*id*(*j*)_ is a random effect that represents the interaction of examinee *i* with testlet *d*(*j*) [*d*(*j*) denotes the testlet *d* contains item *j*]. Further, Li et al. (2006) propose a general two parameter normal ogive testlet response theory (2PNOTRT) model from the perspective of multidimensionality. Each item response in the multidimensional model depends on both the primary dimension and the secondary testlet dimensions. Under the 2PNOTRT model, the basic form of probit link function is expressed as Φ[*a*_*j*1_θ_*i*_ − *t*_*j*_ + *a*_*j*2_η_*id*(*j*)_], where *t*_*j*_ is a threshold parameter related to the item difficulty. The latent traits underlying examinees' responses to items in testlets consist of general ability θ and several secondary dimensions, one for each testlet. Item parameters *a*_*j*1_ and *a*_*j*2_ indicate the discriminating power of an item with respect to the primary ability θ and the secondary dimension η_*d*_, respectively. Because the secondary dimension η_*id*(*j*)_ is a random effect that represents the interaction of examinee *i* with testlet *d*(*j*), it is believed that the loading of the secondary dimensions η_*d*_ should be the discriminating power of the testlet with respect to it, and it should be related to the discrimination parameters of the items in the testlet with respect to the intended ability, θ. The above two testlet effect models are constructed in the framework of probit link function. On this basis, Zhan et al. (2014) propose the concept of within-item multidimensional testlet effect. In this paper, we introduce a new item parameter as a testlet discrimination parameter and propose a new two parameter logistic testlet model in the framework of logit link function for dichotomously scored items, as detailed in the next section. Moreover, testlet response theory modeling has also been extended to the other field of educational and psychological measurement such as large-scale language assessments (Rijmen, 2010; Zhang, 2010; Eckes, 2014), hierarchical data analysis (Jiao et al., 2005, 2013), cognitive diagnostic assessments (Zhan et al., 2015, 2018).

One of the most commonly used estimation methods for the above-mentioned testlet effect models is the marginal maximum likelihood method via the expectation-maximization (EM; Dempster et al., 1977) algorithm (Bock and Aitkin, 1981; Mislevy, 1986; Glas et al., 2000; Wang and Wilson, 2005b). The ability parameters and testlet effects are viewed as unobserved data (latent variables), and then we can find the maximum of a complete data likelihood (the responses and unobserved data) marginalized over unobserved data. However, the marginal maximum likelihood estimation of testlet models has been hampered by the fact that the computations often involve analytically intractable high dimensional integral and hence it is hard to find the maximum likelihood estimate of the parameters. More specifically, when the integrals over latent variable distributions are evaluated using Gaussian quadrature (Bock and Aitkin, 1981), the number of calculations involved increases exponentially with the number of latent variable dimensions. Even though the number of quadrature points per dimension can be reduced when using adaptive Gaussian quadrature (Pinheiro and Bates, 1995), the total number of points again increases exponentially with the number of dimensions. In addition, when the EM algorithm is employed to compute marginal maximum likelihood estimates with unobserved data, the convergence of EM algorithm can be very slow whenever there is a large fraction of unobserved data, and the estimated information matrix is not a direct by product of maximization.

An alternative method is to use a fully Bayesian formulation, coupled with a Markov Chain Monte Carlo (MCMC) procedure to estimate the testlet model parameters (e.g., Wainer et al., 2000, 2007). The Bayesian method, including Metropolis-Hastings algorithm (Metropolis et al., 1953; Hastings, 1970; Tierney, 1994; Chib and Greenberg, 1995; Chen et al., 2000) and Gibbs algorithm (Geman and Geman, 1984; Tanner and Wong, 1987; Albert, 1992), has some significant advantages over classical statistical analysis. It allows meaningful assessments in confidence regions, incorporates prior knowledge into the analysis, yields more precise estimators (provided the prior knowledge is accurate), and follows the likelihood and sufficiency principles. In this current study, an effective slice-Gibbs sampling algorithm (Lu et al., 2018) in the framework of Bayesian is used to estimate the model parameters. The slice-Gibbs sampling, as the name suggests, can be conceived of an extension of Gibbs algorithm. The sampling process consists of two parts. One part is the slice algorithm (Damien et al., 1999; Neal, 2003; Bishop, 2006; Lu et al., 2018), which samples the two parameter logistic testlet effect models from the truncated full conditional posterior distribution by introducing the auxiliary variables. The other part is Gibbs algorithm which updates variance parameters based on the sampled values from the two parameter logistic testlet effect models. The motivation for this sampling algorithm is manifold. First, the slice-Gibbs sampling algorithm is a fully Bayesian method, which averts to calculate multidimensional numerical integration compared with the marginal maximum likelihood method. Second, the slice algorithm has the advantage of a flexible prior distribution being introduced to obtain samples from the full conditional posterior distributions rather than being restricted to using the conjugate distributions, which is required in Gibbs sampling algorithm and limited using the normal ogive framework (Tanner and Wong, 1987; Albert, 1992; Bradlow et al., 1999; Wainer et al., 2000; Fox and Glas, 2001; Fox, 2010; Tao et al., 2013). The detailed discussions about the informative priors and non-informative priors of item parameters are shown in the simulation 2. Third, it is known that the Metropolis-Hasting algorithm (Metropolis et al., 1953; Hastings, 1970; Tierney, 1994; Chib and Greenberg, 1995; Chen et al., 2000) severely depends on the standard deviation (tuning parameter) of the proposal distributions, and it is sensitive to step size. More specifically, if the step size is too small random walk, the chain will take longer to traverse the support of the target density; If the step size is too large there is great inefficiency due to a high rejection rate. However, the slice algorithm automatically tunes the step size to match the local shape of the target density and draws the samples with acceptance probability equal to one. Thus, it is easier and more efficient to implement.

The remainder of this article is organized as follows. Section 2 describes the two parameter logistic testlet effect model, the prior assumptions and model identifications. A detailed description of the slice-Gibbs sampling algorithm and Bayesian model assessment criteria are presented in section 2. In section 3, three simulation studies are given, the first of which considers the performances of parameter recovery using the slice-Gibbs algorithm under different design conditions. In the second simulation, the prior sensitivity of the the slice-Gibbs sampling algorithm is assessed using the simulated data. In the third simulation, based on the Markov chain Monte Carlo (MCMC) output, two Bayesian model assessment methods are used to evaluate the model fit. In section 5, an empirical example is analyzed in detail to further demonstrate the applicability of the testlet structure models and the validity of the slice-Gibbs sampling algorithm. At last, we conclude with a few summary remarks in section 6.

## 2. The New Two Parameter Logistic Testlet Model and Prior Assumptions

The new two parameter logistic testlet model (N2PLTM):

(1) $$\begin{array}{l} p_{ij}=p\left(y_{ij}=1|\theta_{i},a_{j},b_{j},\eta_{id\left(j\right)}\right)\\ \;=\frac{\exp\left[a_{j}\left(\theta_{i}-b_{j}\right)+\alpha_{d\left(j\right)}\eta_{id\left(j\right)}\right]}{1+\exp\left[a_{j}\left(\theta_{i}-b_{j}\right)+\alpha_{d\left(j\right)}\eta_{id\left(j\right)}\right]}, \end{array}$$

In Equation (1), *i* = 1, …, *n*. indicates persons. Suppose a text contains *J* items, items in such tests are grouped into *K*(1 ≤ *K* ≤ *J*) mutually exclusive and exhaustive testlets. Denote testlet *d* containing item by *d*(*j*) and the size of each testlet by *n*_*k*_(1 ≤ *k* ≤ *K*) which can be written as with *d*(1) and *d*(*J*) = *K*. *y*_*ij*_ represents the response of the *i*th examinee answering the *j*th item, and the correct response probability is expressed as *p*_*ij*_. And θ_*i*_ denotes ability parameter for the *i*th examinee. *a*_*j*_ is the discrimination parameter of the item *j*. *b*_*j*_ denotes the difficulty parameter of the item *j*, and $\alpha_{d\left(j\right)}=\underset{j\in S_{d\left(j\right)}}{\sum }\frac{a_{j}}{n_{d\left(j\right)}}$ is the testlet discrimination parameter where *n*_*d*(*j*)_ is the numbers of items in testlet (testlet *d* contains item *j*) and *S*_*d*(*j*)_ is the set of the serial numbers of item in the testlet. The purpose of using the testlet discrimination parameter is to consider the interaction between the discrimination parameters for all *S*_*d*(*j*)_ items in the same testlet and the testlet effect, rather than just examining the influence of the *j*th item discrimination parameter on the testlet effect for the traditional testlet models. The random effect η_*id*(*j*)_ represents the interaction of individual *i* with testlet *d*(*j*). It can be interpreted as a random shift in individuals' ability or another ability dimension (Li et al., 2006). The following priors and hyper-priors are used to estimate the parameters of N2PLTM. The latent ability θ and the testlet effect η are assumed to be independently and normally distributed under the testlet model. That is, $\eta^{*}={\left(\theta_{i},\eta_{i1},\ldots ,\eta_{iK}\right)}^{\prime }$ has a multivariate normal distribution *N*(**μ**, **Σ**), where **μ** is mean vector, **Σ** is a diagonal matrix, $\Sigma =diag\left(\sigma_{\theta }^{2},\sigma_{\eta_{1}}^{2},\ldots ,\sigma_{\eta_{K}}^{2}\right)$. The variances of η_*ik*_ (*k* = 1, 2, …, *K*), which can be allowed to vary across testlets, indicate the amount of local dependence in each testlet. If the variance of η_*ik*_ is zero, the items within the testlet can be considered conditionally independent. As the variance increases, the amount of local dependence increases. The priors to the discrimination parameters are set from truncated normal priors, $N\left(\mu_{a},\sigma_{a}^{2}\right)\text{I}\left(0,+\infty \right)$, where I(0, +∞) denotes the indicator function that the values range from zero to infinity, and the difficulty parameters are assumed to follow the normal distribution, $b_{k}\sim N\left(\mu_{b},\sigma_{b}^{2}\right)$. In addition, the hyper-priors for $\sigma_{a}^{2}$, $\sigma_{b}^{2}$ and $\sigma_{\eta_{k}}^{2}\left(k=1,2,\ldots ,K\right)$ are assumed to follow inverse Gamma distribution with shape parameter *v* and scale parameter τ. Let **Ω** = (**θ**, *****a*****, *****b*****, **η**) represents the collection of the unknown parameters in model (1), where $\theta ={\left(\theta_{1},\ldots ,\theta_{n}\right)}^{\prime }$, $a={\left(a_{1},\ldots ,a_{J}\right)}^{\prime }$, $b={\left(b_{1},\ldots ,b_{J}\right)}^{\prime }$ and $\eta ={\left(\eta_{1d\left(1\right)},\ldots .,\eta_{d\left(J\right)}\right)}^{\prime }.$ The joint posterior distribution of **Ω** given the data is represented by

(2) $$\begin{array}{l} p\left(\Omega |Y\right)\propto \overset{n}{\underset{i=1}{\prod }}\overset{J}{\underset{j=1}{\prod }}p\left(y_{ij}|\theta_{i},a_{j},b_{j},\eta_{id\left(j\right)}\right)p\left(\theta_{i}\right)\\ \;p\left(a_{j}|\mu_{a},\sigma_{a}^{2}\right)\text{I}\left(a_{j}>0\right)p\left(b_{j}|\mu_{b},\sigma_{b}^{2}\right)\\ \;\times p\left(\sigma_{a}^{2}\right)p\left(\sigma_{b}^{2}\right)p\left(\eta_{id\left(j\right)}|\mu_{\eta },\sigma_{\eta_{d\left(j\right)}}^{2}\right)p\left(\sigma_{\eta_{d\left(j\right)}}^{2}\right)\\ \;\propto \left\{\overset{n}{\underset{i=1}{\prod }}\overset{J}{\underset{j=1}{\prod }}\left[p_{ij}^{y_{ij}}{\left(1-p_{ij}\right)}^{1-y_{ij}}\right]\right\}\left[\overset{n}{\underset{i=1}{\prod }}\exp\left(-\frac{\theta_{i}^{2}}{2}\right)\right]\\ \;{\left(\sigma_{a}^{2}\sigma_{b}^{2}\right)}^{-\frac{J}{2}}\overset{J}{\underset{j=1}{\prod }}\exp\left[-\frac{{\left(a_{j}-\mu_{a}\right)}^{2}}{2\sigma_{a}^{2}}\right]\\ \;\times \exp\left[-\frac{{\left(b_{j}-\mu_{b}\right)}^{2}}{2\sigma_{b}^{2}}\right]\text{I}\left(a_{j}>0\right){\left(\sigma_{a}^{2}\right)}^{-\left(v_{1}+1\right)}\\ \;{\left(\sigma_{b}^{2}\right)}^{-\left(v_{2}+1\right)}\exp\left[-\frac{\tau_{1}}{\sigma_{a}^{2}}-\frac{\tau_{2}}{\sigma_{b}^{2}}\right]\\ \;\times \overset{n}{\underset{i=1}{\prod }}\overset{J}{\underset{j=1}{\prod }}\exp\left(-\frac{\eta_{id\left(j\right)}^{2}}{2\sigma_{\eta_{d\left(j\right)}}^{2}}\right){\left(\sigma_{\eta_{d\left(j\right)}}^{2}\right)}^{-\left(v_{3}+1\right)}\\ \;\exp\left(-\frac{\tau_{3}}{\sigma_{\eta_{d\left(j\right)}}^{2}}\right). \end{array}$$

### 2.1. Model Identifications

In Equation 1, the linear part of the testlet effect model, *a*_*j*_(θ_*i*_ − *b*_*j*_) + α_*d*(*j*)_η_*id*(*j*)_, can be rewritten as follows

$$a_{j}\left(\theta_{i}-b_{j}+\frac{\eta_{id\left(j\right)}}{n_{d\left(j\right)}}\right)+\frac{\underset{k\in S_{d\left(j\right)}-\left\{j\right\}}{\sum }a_{k}}{n_{d\left(j\right)}}\eta_{id\left(j\right)},$$

where the testlet discrimination α_*d*(*j*)_ consists of the discrimination parameters *a*_*j*_. That is, $\alpha_{d\left(j\right)}=\underset{j\in S_{d\left(j\right)}}{\sum }\frac{a_{j}}{n_{d\left(j\right)}}$, and *k* ∈ *S*_*d*(*j*)_ − {*j*} means that *k* belongs to the set *S*_*d*(*j*)_ excluding the index *j*. To eliminate the trade offs among the ability θ, difficulty parameter *b* and testlet effect η_*id*(*j*)_ in location, we fix the mean population level of ability to zero and restrict a item difficulty parameter to zero. Meanwhile, to eliminate the trade off between the ability θ and the discrimination parameter *a* in scale, we need restrict the variance population level of ability to one. However, *a*_*j*_*b*_*j*_, $a_{j}\frac{\eta_{id\left(j\right)}}{n_{d\left(j\right)}}$ and $\frac{\underset{k\in S_{d\left(j\right)}-\left\{j\right\}}{\sum }a_{k}}{n_{d\left(j\right)}}\eta_{id\left(j\right)}$ still have the trade offs in scale. In fact, we only need fix a item discrimination parameter to one. In summary, the required identification conditions are as follows:

$$\theta_{i}\sim N\left(0,1\right),\;a_{1}=1\text{ and }b_{1}=0.$$

Several identification restriction methods of two parameter IRT models have been widely used. The identification restrictions of our model are based on the following methods.

(1) To fix the mean population level of ability to zero and the variance population level of ability to one (Lord and Novick, 1968; Bock and Aitkin, 1981; Fox and Glas, 2001; Fox, 2010). That is, θ ~ *N*(0, 1);
(2) To fix the item difficulty parameter to a specific value, most often zero, and restrict the discrimination parameter to a specific value, most often one (Fox and Glas, 2001; Fox, 2010). That is, *b*_1_ = 0 and *a*_1_ = 1.

## 3. Bayesian Inferences

### 3.1. Slice-Gibbs Algorithm to Estimate Model Parameters

The motivation for the slice-Gibbs sampling algorithm is that the inferred samples can easily be drawn from the full conditional distribution by introducing the auxiliary variables. Before giving the specific Bayesian sampling process, we give the definition of auxiliary and its role in the sampling process. Auxiliary variables are variables that can help to make estimates on incomplete data, while they are not part of the main analysis. Basically, the auxiliary variables are latent unknown parameters without any direct interpretation which are introduced for technical/simulation reasons or for the reason of making an analytically intractable distribution tractable. Within the Bayesian framework, in the method of auxiliary variables, realizations from a complicated distribution can be obtained by augmenting the variables of interest by one or more additional variables such that the full conditionals are tractable and easy to simulate from. The construction of sampling algorithms via the introduction of auxiliary variable received much attention since it resulted in both simple and fast algorithms (Tanner and Wong, 1987; Higdon, 1998; Meng and van Dyk, 1999; Fox, 2010).

For each of the response variable *y*_*ij*_, we introduce two mutually independent random auxiliary variables λ_*ij*_ and φ_*ij*_. The random variables λ_*ij*_ and φ_*ij*_ are assumed to follow a Uniform (0,1). The following two cases must be satisfied.

**Case 1**: When *y*_*ij*_ = 1, an equivalent condition for *y*_*ij*_ = 1 is the indicator function I(0 < λ_*ij*_ ≤ *p*_*ij*_) must be equal to 1, as opposed to I(0 < φ_*ij*_ ≤ *q*_*ij*_) is set to 0, where *q*_*ij*_ = 1 − *p*_*ij*_. In addition, if the joint distribution (λ_*ij*_ and *p*_*ij*_) integrate out the auxiliary variables λ_*ij*_, the obtained marginal distribution is just equal to the correct response probability of the *i*th individual answering the *j*th item.

**Case 2**: Similarly, when *y*_*ij*_ = 0, an equivalent condition for *y*_*ij*_ = 0, that is, the indicator function I(0 < φ_*ij*_ ≤ *q*_*ij*_) must be equal to 1, as opposed to is I(0 < λ_*ij*_ ≤ *p*_*ij*_) set to 0.

Therefore, the joint posterior distribution based on the auxiliary variables is given by

(3) $$\begin{array}{l} p\left(\Omega ,\lambda ,\varphi |Y\right)\propto \prod_{i=1}^{n}\prod_{j=1}^{J}\left[\left(y_{ij}=1\right)\text{I}\left(0<\lambda_{ij}\leq p_{ij}\right)\right]\\ \;+\text{I}\left(y_{ij}=0\right)\text{I}\left(0<\varphi_{ij}\leq q_{ij}\right)\\ \;\times {\left(\sigma_{a}^{2}\sigma_{b}^{2}\right)}^{-\frac{J}{2}}\prod_{j=1}^{J}\exp\left[-\frac{{\left(a_{j}-\mu_{a}\right)}^{2}}{2\sigma_{a}^{2}}-\frac{{\left(b_{j}-\mu_{b}\right)}^{2}}{2\sigma_{b}^{2}}\right]\\ \text{ I}\left(a_{j}>0\right)\left[\prod_{j=1}^{n}\exp\left(-\frac{\theta_{i}^{2}}{2}\right)\right]\\ \;\times {\left(\sigma_{a}^{2}\right)}^{-\left(v_{1}+1\right)}{\left(\sigma_{b}^{2}\right)}^{-\left(v_{2}+1\right)}\exp\left[-\frac{\tau_{1}}{\sigma_{a}^{2}}-\frac{\tau_{2}}{\sigma_{b}^{2}}\right]\\ \;\times \prod_{i=1}^{n}\prod_{j=1}^{J}\exp\left(-\frac{\eta_{id(j)}^{2}}{2\sigma_{\eta_{d(j)}}^{2}}\right){\left(\sigma_{\eta_{d(j)}}^{2}\right)}^{-\left(v_{3}+1\right)}\\ \;\exp\left(-\frac{\tau_{3}}{\sigma_{\eta_{d(j)}}^{2}}\right). \end{array}$$

We find that the Equation (2) can be obtained by taking expectations about the auxiliary variables for the Equation (3). Each step of the algorithm needs to satisfy the Equation (3). The detailed slice-Gibbs sampling algorithm is given by

**Step 1**: Sample the auxiliary variables λ_*ij*_ and φ_*ij*_ given the response variable *****Y***** and the parameters **Ω**. The full conditional posterior distributions can be written as

(4) $$\begin{array}{l} \begin{array}{cc} \lambda_{ij}|Y,\;\Omega \sim \text{Uniform}\left(0,\;p_{ij}\right), & \text{if }y_{ij}=1,\\ \varphi_{ij}|Y,\;\Omega \sim \text{Uniform}\left(0,\;q_{ij}\right), & \text{if }y_{ij}=0. \end{array} \end{array}$$

**Step 2**: Sample the discrimination parameter *a*_*j*_. The prior of the discrimination parameters is $N\left(\mu_{a},\sigma_{a}^{2}\right)\text{I}\left(0,+\infty \right)$. According to the Equation (3), for all *i*, if 0 < λ_*ij*_ ≤ *p*_*ij*_, $\left(\theta_{i}-b_{j}+\frac{\eta_{id\left(j\right)}}{n_{d\left(j\right)}}\right)>0$ or 0 < φ_*ij*_ ≤ *q*_*ij*_, $\left(\theta_{i}-b_{j}+\frac{\eta_{id\left(j\right)}}{n_{d\left(j\right)}}\right)<0$. The following inequalities are established

$$a_{j}\left(\theta_{i}-b_{j}\right)+\alpha_{d\left(j\right)}\eta_{id\left(j\right)}\geq \log\left(\frac{\lambda_{ij}}{1-\lambda_{ij}}\right),$$

Or equivalently,

$$a_{j}\geq {\left(\theta_{i}-b_{j}+\frac{\eta_{id\left(j\right)}}{n_{d\left(j\right)}}\right)}^{-1}\left[\log\left(\frac{\lambda_{ij}}{1-\lambda_{ij}}\right)-\frac{\underset{k\in S_{d\left(j\right)}-\left\{j\right\}}{\sum }a_{k}}{n_{d\left(j\right)}}\eta_{id\left(j\right)}\right],$$

And,

$$a_{j}\left(\theta_{i}-b_{j}\right)+\alpha_{d\left(j\right)}\eta_{id\left(j\right)}\geq \log\left(\frac{1-\varphi_{ij}}{\varphi_{ij}}\right),$$

Or equivalently,

$$a_{j}\geq {\left(\theta_{i}-b_{j}+\frac{\eta_{id\left(j\right)}}{n_{d\left(j\right)}}\right)}^{-1}\left[\log\left(\frac{1-\varphi_{ij}}{\varphi_{ij}}\right)-\frac{\underset{k\in S_{d\left(j\right)}-\left\{j\right\}}{\sum }a_{k}}{n_{d\left(j\right)}}\eta_{id\left(j\right)}\right].$$

Similarly, for all *i*, if 0 < λ_*ij*_ ≤ *p*_*ij*_, $\left(\theta_{i}-b_{j}+\frac{\eta_{id\left(j\right)}}{n_{d\left(j\right)}}\right)<0$ or 0 < φ_*ij*_ ≤ *q*_*ij*_, $\left(\theta_{i}-b_{j}+\frac{\eta_{id\left(j\right)}}{n_{d\left(j\right)}}\right)>0$. The following inequalities are established

$$a_{j}\left(\theta_{i}-b_{j}\right)+\alpha_{d\left(j\right)}\eta_{id\left(j\right)}\geq \log\left(\frac{\lambda_{ij}}{1-\lambda_{ij}}\right),$$

Or equivalently,

$$a_{j}\leq {\left(\theta_{i}-b_{j}+\frac{\eta_{id\left(j\right)}}{n_{d\left(j\right)}}\right)}^{-1}\left[\log\left(\frac{\lambda_{ij}}{1-\lambda_{ij}}\right)-\frac{\underset{k\in S_{d\left(j\right)}-\left\{j\right\}}{\sum }a_{k}}{n_{d\left(j\right)}}\eta_{id\left(j\right)}\right],$$

And,

$$a_{j}\left(\theta_{i}-b_{j}\right)+\alpha_{d\left(j\right)}\eta_{id\left(j\right)}\geq \log\left(\frac{1-\varphi_{ij}}{\varphi_{ij}}\right),$$

Or equivalently,

$$a_{j}\leq {\left(\theta_{i}-b_{j}+\frac{\eta_{id\left(j\right)}}{n_{d\left(j\right)}}\right)}^{-1}\left[\log\left(\frac{1-\varphi_{ij}}{\varphi_{ij}}\right)-\frac{\underset{k\in S_{d\left(j\right)}-\left\{j\right\}}{\sum }a_{k}}{n_{d\left(j\right)}}\eta_{id\left(j\right)}\right].$$

Let

$$\begin{array}{l} \Delta_{j}=\left\{i|0<\lambda_{ij}\leq p_{ij},\left(\theta_{i}-b_{j}+\frac{\eta_{id\left(j\right)}}{n_{d\left(j\right)}}\right)>0\right\},\\ G_{j}=\left\{i|0<\varphi_{ij}\leq p_{ij},\left(\theta_{i}-b_{j}+\frac{\eta_{id\left(j\right)}}{n_{d\left(j\right)}}\right)<0\right\},\\ \nabla_{j}=\left\{i|0<\lambda_{ij}\leq p_{ij},\left(\theta_{i}-b_{j}+\frac{\eta_{id\left(j\right)}}{n_{d\left(j\right)}}\right)<0\right\},\\ \Lambda_{j}=\left\{i|0<\varphi_{ij}\leq p_{ij},\left(\theta_{i}-b_{j}+\frac{\eta_{id\left(j\right)}}{n_{d\left(j\right)}}\right)>0\right\}. \end{array}$$

When given the response variable *****Y*****, the auxiliary variable **λ**, **φ** and other parameters **Ω**_1_ (all of the parameters except *a*_*j*_), the full conditional distribution is represented by

(5) $$\begin{array}{l} a_{j}|\lambda ,\;\varphi ,\;\Omega_{1}\sim N\left(\mu_{a},\sigma_{a}^{2}\right)\text{I}\left(0<a_{j}^{L}\leq a_{j}\leq a_{j}^{U}\right). \end{array}$$

In Equation (5),

$$\begin{array}{l} a_{j}^{L}=\max\{\underset{i\in \Delta_{j}}{\max}{\left(\theta_{i}-b_{j}+\frac{\eta_{id\left(j\right)}}{n_{d\left(j\right)}}\right)}^{-1}\\ \;\left[\log\left(\frac{\lambda_{ij}}{1-\lambda_{ij}}\right)-\frac{\sum_{k\in S_{d\left(j\right)}-\{j\}}a_{k}}{n_{d(j)}}\eta_{id(j)}\right],\\ \underset{i\in G_{j}}{\max}{\left(\theta_{i}-b_{j}+\frac{\eta_{id\left(j\right)}}{n_{d\left(j\right)}}\right)}^{-1}\left[\log\left(\frac{1-\varphi_{ij}}{\varphi_{ij}}\right)-\frac{\sum_{k\in S_{d\left(j\right)}-\{j\}}a_{k}}{n_{d(j)}}\eta_{id(j)}\right]\}. \end{array}$$

And

$$\begin{array}{l} a_{j}^{U}=\min\{\underset{i\in \nabla_{j}}{\min}{\left(\theta_{i}-b_{j}+\frac{\eta_{id\left(j\right)}}{n_{d\left(j\right)}}\right)}^{-1}\\ \;\left[\log\left(\frac{\lambda_{ij}}{1-\lambda_{ij}}\right)-\frac{\sum_{k\in S_{d\left(j\right)}-\{j\}}a_{k}}{n_{d(j)}}\eta_{id(j)}\right],\\ \underset{i\in \Lambda_{j}}{\min}{\left(\theta_{i}-b_{j}+\frac{\eta_{id\left(j\right)}}{n_{d\left(j\right)}}\right)}^{-1}\left[\log\left(\frac{1-\varphi_{ij}}{\varphi_{ij}}\right)-\frac{\sum_{k\in S_{d\left(j\right)}-\{j\}}a_{k}}{n_{d(j)}}\eta_{id(j)}\right]\}. \end{array}$$

**Step 3**: Sample the difficulty parameter *b*_*j*_. The prior of the difficulty parameters is $N\left(\mu_{b},\sigma_{b}^{2}\right)$. According to the Equation (3), for ∀*i*, if we have 0 < λ_*ij*_ ≤ *p*_*ij*_, the following inequalities are established,

$$a_{j}\left(\theta_{i}-b_{j}\right)+\alpha_{d\left(j\right)}\eta_{id\left(j\right)}\geq \log\left(\frac{\lambda_{ij}}{1-\lambda_{ij}}\right),$$

Or equivalently,

$$b_{j}\leq \theta_{i}-\frac{1}{a_{j}}\left[\log\left(\frac{\lambda_{ij}}{1-\lambda_{ij}}\right)-\alpha_{d\left(j\right)}\eta_{id\left(j\right)}\right].$$

Similarly, for all *i*, if 0 < φ_*ij*_ ≤ *q*_*ij*_, the following inequalities are established

$$a_{j}\left(\theta_{i}-b_{j}\right)+\alpha_{d\left(j\right)}\eta_{id\left(j\right)}\geq \log\left(\frac{1-\varphi_{ij}}{\varphi_{ij}}\right),$$

Or equivalently,

$$b_{j}\leq \theta_{i}-\frac{1}{a_{j}}\left[\log\left(\frac{1-\varphi_{ij}}{\varphi_{ij}}\right)-\alpha_{d\left(j\right)}\eta_{id\left(j\right)}\right].$$

Let *D*_*j*_ = {*i*|*y*_*ij*_ = 1, 0 < λ_*ij*_ ≤ *p*_*ij*_}, *E*_*j*_ = {*i*|*y*_*ij*_ = 0, 0 < φ_*ij*_ ≤ *q*_*ij*_}. Thus, given the response variable *****Y*****, the auxiliary variable **λ**, **φ** and other parameters **Ω**_2_ (all of the parameters except *b*_*j*_). The full conditional posterior distribution is given by

(6) $$\begin{array}{l} b_{j}|\lambda ,\;\varphi ,\;\Omega_{2}\sim N\left(\mu_{b},\sigma_{b}^{2}\right)\text{I}\left(b_{j}^{L}\leq b_{j}\leq b_{j}^{U}\right), \end{array}$$

In Equation (6),

$$b_{j}^{L}=\underset{i\in E_{j}}{\max}\left\{\theta_{i}-\frac{1}{a_{j}}\left[\log\left(\frac{1-\varphi_{ij}}{\varphi_{ij}}\right)-\alpha_{d\left(j\right)}\eta_{id\left(j\right)}\right]\right\},$$

And

$$b_{j}^{U}=\underset{i\in D_{j}}{\min}\left\{\theta_{i}-\frac{1}{a_{j}}\left[\log\left(\frac{\lambda_{ij}}{1-\lambda_{ij}}\right)-\alpha_{d\left(j\right)}\eta_{id\left(j\right)}\right]\right\}.$$

**Step 4**: Sample the latent ability θ_*i*_, the prior of the latent ability is assumed to follow a normal distribution with mean μ_θ_ and variance $\sigma_{\theta }^{2}$. Given the response variable *****Y*****, the auxiliary variable **λ**, **φ** and other parameters **Ω**_3_ (all of the parameters except θ_*i*_). The full conditional posterior distribution of θ_*i*_ is

(7) $$\begin{array}{l} \theta_{i}|\lambda ,\;\varphi ,\;\Omega_{3},Y\sim N\left(\mu_{\theta },\sigma_{\theta }^{2}\right)\text{I}\left(\theta_{i}^{L}\leq \theta_{i}\leq \theta_{i}^{U}\right), \end{array}$$

In Equation (7),

$$\begin{array}{l} \theta_{i}^{L}=\underset{j\in C_{i}}{\max}\left\{\frac{1}{a_{j}}\left[\log\left(\frac{\lambda_{ij}}{1-\lambda_{ij}}\right)-\alpha_{d\left(j\right)}\eta_{id\left(j\right)}\right]+b_{j}\right\},\\ \text{ where }C_{i}=\left\{j|y_{ij}=1,\;0<\lambda_{ij}\leq p_{ij}\right\},\\ \theta_{i}^{U}=\underset{j\in B_{i}}{\min}\left\{\frac{1}{a_{j}}\left[\log\left(\frac{1-\varphi_{ij}}{\varphi_{ij}}\right)-\alpha_{d\left(j\right)}\eta_{id\left(j\right)}\right]+b_{j}\right\},\\ \text{ where }B_{i}=\left\{j|y_{ij}=0,\;0<\varphi_{ij}\leq q_{ij}\right\}. \end{array}$$

**Step 5**: Sample the testlet random effect η_*id*(*j*)_. Assuming that the *j*th term comes from the *k*th testlet [i.e., *d*(*j*) = *k*] and the order of the terms in the *k*th testlet is form *j*_*k*_ to *n*_*k*_ + *j*_*k*_ − 1. Then, the joint posterior distribution can be rewritten as

$$\begin{array}{l} p\left(\Omega ,\lambda ,\varphi |Y\right)\propto \prod_{i=1}^{n}\prod_{k=1}^{K}\prod_{j=j_{k}}^{n_{k}+j_{k}-1}[\text{I}\left(y_{ij}=1\right)\text{I}\left(0<\lambda_{ij}\leq p_{ij}^{*}\right)\\ \;+\text{I}\left(y_{ij}=0\right)\text{I}\left(0<\varphi_{ij}\leq q_{ij}^{*}\right)\\ \;\times {\left(\sigma_{a}^{2}\sigma_{b}^{2}\right)}^{-\frac{J}{2}}\prod_{j=1}^{J}\exp[-\frac{{\left(a_{j}-\mu_{a}\right)}^{2}}{2\sigma_{a}^{2}}\\ \;-\frac{{\left(b_{j}-\mu_{b}\right)}^{2}}{2\sigma_{b}^{2}}]\text{I}\left(a_{j}>0\right)\left[\prod_{i=1}^{n}\left(-\frac{\theta_{i}^{2}}{2}\right)\right]\\ \times {\left(\sigma_{a}^{2}\right)}^{-\left(v_{1}+1\right)}{\left(\sigma_{b}^{2}\right)}^{-\left(v_{2}+1\right)}\exp\left[-\frac{\tau_{1}}{\sigma_{a}^{2}}-\frac{\tau_{2}}{\sigma_{b}^{2}}\right]\\ \times \prod_{i=1}^{n}\prod_{j=1}^{J}\exp\left(-\frac{\eta_{ik}^{2}}{2\sigma_{\eta_{k}}^{2}}\right){\left(\sigma_{\eta_{k}}^{2}\right)}^{-\left(v_{3}+1\right)}\exp\left(-\frac{\tau_{3}}{\sigma_{\eta_{k}}^{2}}\right). \end{array}$$

where $p_{ij}^{*}=\frac{\exp\left[a_{j}\left(\theta_{i}-b_{j}\right)+\alpha_{k}\eta_{ik}\right]}{1+\exp\left[a_{j}\left(\theta_{i}-b_{j}\right)+\alpha_{k}\eta_{ik}\right]},$ $q_{ij}^{*}=1-p_{ij}^{*}.$ The prior of the testlet random effect η_*ik*_ is assumed to follow a normal distribution with mean μ_η_ and variance $\sigma_{\eta }^{2}$. Given the response variable *****Y*****, the auxiliary variable **λ**, **φ** and other parameters **Ω**_4_ (all of the parameters except η_*ik*_). The full conditional distribution of η_*ik*_ is given by

(8) $$\begin{array}{l} \eta_{ik}|\lambda ,\;\varphi ,\;\Omega_{4},Y\sim N\left(\mu_{\eta },\sigma_{\eta }^{2}\right)\text{I}\left(\eta_{ik}^{L}\leq \eta_{ik}\leq \eta_{ik}^{U}\right), \end{array}$$

In Equation (8),

$$\begin{array}{l} \eta_{ik}^{L}=\frac{1}{\alpha_{k}}\left[\log\left(\frac{\lambda_{ij}}{1-\lambda_{ij}}\right)-a_{j}\left(\theta_{i}-b_{j}\right)\right],\text{and}\\ \eta_{ik}^{U}=\frac{1}{\alpha_{k}}\left[\log\left(\frac{1-\varphi_{ij}}{\varphi_{ij}}\right)-a_{j}\left(\theta_{i}-b_{j}\right)\right]. \end{array}$$

**Step 6**: Sample the variance parameter $\sigma_{a}^{2}$, the variance is assumed to follow a Inverse-Gamma(*v*_1_, τ_1_) hyper prior. Given the discrimination parameters *****a*****, the hyper parameters *v*_1_ and τ_1_. The full conditional posterior distribution of $\sigma_{a}^{2}$ is given by

$$\begin{array}{l} p\left(\sigma_{a}^{2}|a,v_{1},\tau_{1}\right)\propto p\left(a|\mu_{a},\sigma_{a}^{2}\right)p\left(\sigma_{a}^{2}\right)\\ \;\propto |\sigma_{a}^{2}{|}^{-\frac{J}{2}}\exp\left\{-\frac{\overset{J}{\underset{j=1}{\sum }}{\left(a_{j}-\mu_{a}\right)}^{2}}{2\sigma_{a}^{2}}\right\}\\ \;|\sigma_{a}^{2}{|}^{-\left(v_{1}+1\right)}\exp\left\{-\frac{\tau_{1}}{\sigma_{a}^{2}}\right\}. \end{array}$$

Thus,

$$\sigma_{a}^{2}|a,v_{1},\tau_{1}\sim \text{Inverse}-\text{Gamma}\left(\frac{J}{2}+v_{1},\frac{\overset{J}{\underset{j=1}{\sum }}{\left(a_{j}-\mu_{a}\right)}^{2}}{2}+\tau_{1}\right).$$

**Step 7**: Sample the variance parameter $\sigma_{b}^{2}$, the variance is assumed to follow a Inverse-Gamma(*v*_2_, τ_2_) hyper prior. Given the difficulty parameters *****b*****, the hyper parameters *v*_2_ and τ_2_. The full conditional posterior distribution of $\sigma_{b}^{2}$ is given by

$$\begin{array}{l} p\left(\sigma_{b}^{2}|b,v_{2},\tau_{2}\right)\propto p\left(b|\mu_{b},\sigma_{b}^{2}\right)p\left(\sigma_{b}^{2}\right)\\ \;\propto |\sigma_{b}^{2}{|}^{-\frac{J}{2}}\exp\left\{-\frac{\overset{J}{\underset{j=1}{\sum }}{\left(b_{j}-\mu_{b}\right)}^{2}}{2\sigma_{b}^{2}}\right\}\\ \;|\sigma_{b}^{2}{|}^{-\left(v_{2}+1\right)}\exp\left\{-\frac{\tau_{2}}{\sigma_{b}^{2}}\right\}. \end{array}$$

Thus,

(9) $$\begin{array}{l} \sigma_{b}^{2}|b,v_{2},\tau_{2}\sim \text{Inverse}-\text{Gamma}\left(\frac{J}{2}+v_{2},\frac{\overset{J}{\underset{j=1}{\sum }}{\left(b_{j}-\mu_{b}\right)}^{2}}{2}+\tau_{2}\right). \end{array}$$

**Step 8**: Sample the random effect variance parameter $\sigma_{\eta_{k}}^{2}$, the variance is assumed to follow a Inverse-Gamma (*v*_3_, τ_3_) hyper prior. Given the random effect parameters η, the hyper parameters *v*_3_ and τ_3_. The full conditional posterior distribution of $\sigma_{\eta_{k}}^{2}$ is given by

$$\begin{array}{l} p\left(\sigma_{\eta_{k}}^{2}|\eta ,v_{3},\tau_{3}\right)\propto p\left(\eta |\mu_{\eta },\sigma_{\eta_{k}}^{2}\right)p\left(\sigma_{\eta_{k}}^{2}\right)\\ \;\propto |\sigma_{\eta_{k}}^{2}{|}^{-\frac{n}{2}}\exp\left\{-\frac{\overset{n}{\underset{i=1}{\sum }}{\left(\eta_{ik}-\mu_{\eta }\right)}^{2}}{2\sigma_{\eta_{k}}^{2}}\right\}\\ \;|\sigma_{\eta_{k}}^{2}{|}^{-\left(v_{3}+1\right)}\exp\left\{-\frac{\tau_{3}}{\sigma_{\eta_{k}}^{2}}\right\}. \end{array}$$

Thus,

(10) $$\begin{array}{l} \sigma_{\eta_{k}}^{2}|\eta ,v_{3},\tau_{3}\sim \text{Inverse}-\text{Gamma}\left(\frac{n}{2}+v_{3},\frac{\overset{N}{\underset{i=1}{\sum }}{\left(\eta_{ik}-\mu_{\eta }\right)}^{2}}{2}+\tau_{3}\right). \end{array}$$

### 3.2. Bayesian Model Assessment

Within the framework of Bayesian, Bayes factor has played a major role in assessing the goodness of fit of competing models (Kass and Wasserman, 1995; Gelfand, 1996). It is defined as the ratio of the posterior odds of model 1 to model 2 divided by the prior odds of model 1 to model 2

(11) $$\begin{array}{l} BF=\frac{p\left(M_{1}|y\right)/p\left(M_{2}|y\right)}{p\left(M_{1}\right)/p\left(M_{2}\right)}=\frac{p\left(y|M_{1}\right)}{p\left(y|M_{2}\right)}, \end{array}$$

In Equation (11), *****y***** denotes the observation data, *p*(*M*_*h*_) denotes the model prior likelihood, and *p*(*M*_*h*_|*****y*****) are the marginal likelihoods of the data matrix *****y***** for model *h*, *h* = 1, 2. The Bayes factor (BF) provide a summary of evidence for *M*_1_ compared to *M*_2_. *M*_1_ is supported when BF > 1, and *M*_2_ is supported otherwise. A value of BF between 1 and 3 is considered as minimal evidence for *M*_1_, a value between 3 and 12 as positive evidence for *M*_1_, a value between 12 and 150 as strong evidence for *M*_1_, and a value >150 as very strong evidence (Raftery, 1996). However, one of the obstacles to use of the Bayes factors is the difficulty associated with calculating them. As we known, while the candidate model with high-dimensional parameters are used to fit the data, it is not possible integrate out the all parameters of models to obtain the closed-form expression of marginal distribution. In addition, it are acutely sensitive to the choice of prior distributions. If the use of improper priors for the parameters in alternative models results in Bayes factors that are not well defined. However, numerous approaches have been proposed for model comparison with improper priors (Aitkin, 1991; Gelfand et al., 1992; Berger and Pericchi, 1996; Ando, 2011). In our article, Based on the noninformative priors, a “pseudo-Bayes factor” approach is implemented, which provides a type of approximation to the BF.

#### 3.2.1. Pseudo-Bayes Factor

The pseudo-Bayes factor (PsBF) method (Geisser and Eddy, 1979) overcome BF sensitive to the choice of prior distributions. It can be obtained by calculating the cross-validation predictive densities. Considering *i* = 1, …, *n* individuals response to items. Let *****y*****_−(*ij*)_ be the observed data without the *ij*th observation and let **Ξ** denote all the parameters under the assumed model. The cross-validation predictive density (CVPD) can be defined by

(12) $$\begin{array}{l} p\left(y_{ij}|y_{-\left(ij\right)}\right)=\int p\left(y_{ij}|y_{-\left(ij\right)},\Xi \right)p\left(\Xi |y_{-\left(ij\right)}\right)d\Xi , \end{array}$$

In Equation (12), the density *p*(*y*_*ij*_|*****y*****_ − (*ij*)_) denotes supporting the possibility of values of *y*_*ij*_ when the model is fitted to observations except *y*_*ij*_. According to conditional independence hypothesis, the equation *p*(*y*_*ij*_|*****y*****_−(*ij*)_, **Ξ**) = *p*(*y*_*ij*_|**Ξ**) can be established, the responses on the different items are independent given ability and the responses of the individuals are independent of one another. The Pseudo Bayes factor (PsBF) for comparing two models (*M*_1_ and *M*_2_) is expressed in terms of the product of cross-validation predictive densities and can be written as

(13) $$\begin{array}{l} PsBF=\underset{i,j}{\prod }\frac{p\left(y_{ij}|y_{-\left(ij\right)},M_{1}\right)}{p\left(y_{ij}|y_{-\left(ij\right)},M_{2}\right)}. \end{array}$$

In practice, we can calculate the logarithm of the numerator and denominator of the PsBF and it can be used for comparing different models. The model with a larger PsBF has a better fit of the data. Gelfand and Dey (1994) and Newton and Raftery (1994) proposed an importance sampling to evaluate the marginal likelihood (CVPD) of the data. Given the sample size *R*, *r* = 1, …, *R*, the samples **Ξ**^(*m*)^ from the posterior distribution *p*(**Ξ**|*****y*****_−(*ij*)_) often easily obtained via an MCMC sampler. The estimated likelihood function is

(14) $$\begin{array}{l} \hat{p\left(y_{ij}|y_{-\left(ij\right)}\right)}={\left[\frac{1}{M}\overset{M}{\underset{m=1}{\sum }}\frac{1}{p\left(y_{ij}|\Xi^{\left(m\right)}\right)}\right]}^{-1}\\ \;={\left[\frac{1}{M}\overset{M}{\underset{m=1}{\sum }}\frac{1}{{\left(p_{ij}^{\left(m\right)}\right)}^{y_{ij}}{\left(1-p_{ij}^{\left(m\right)}\right)}^{1-y_{ij}}}\right]}^{-1}. \end{array}$$

#### 3.2.2. The Deviance Information Criteria (DIC)

A model comparison method is often based on a measure of fit and some penalty function based on the number of free parameters for the complexity of the model. Two well-known criteria of model selection based on a deviance fit measure are the Bayesian information criterion (BIC; Schwarz, 1978) and Akaike's information criterion (AIC; Akaike, 1973). These criteria depend on the effective number of parameters in the model as a measure of model complexity. However, in Bayesian hierarchical models, it is not clear how to define the number of parameters due to the prior distribution imposes additional restrictions on the parameter space and reduces its effective dimension. Therefore, Spiegelhalter et al. (2002) proposed the deviance information criterion (DIC) for model comparison when the number of parameters is not clearly defined in hierarchical models. The DIC is defined as the sum of a deviance measure and a penalty term for the effective number of parameters based on a measure of model complexity. This term estimates the number of effective model parameters and equals

(15) $$\begin{array}{l} P_{D}=E_{\Xi |y}\left\{-2\log p\left(y|\Xi \right)\right\}+2\log p\left(y|\hat{\Xi }\right)\\ \;=\bar{D\left(\Xi \right)}-D\left(\hat{\Xi }\right). \end{array}$$

The DIC can be defined as

(16) $$\begin{array}{l} \text{DIC}=\bar{D\left(\Xi \right)}+P_{D}\\ \;=\bar{D\left(\Xi \right)}+\left(\bar{D\left(\Xi \right)}-D\left(\hat{\Xi }\right)\right). \end{array}$$

In Equation (15), **Ξ** is the parameter of interest in the model. The complexity is measured by the effective number of parameters, *P*_*D*_. $\bar{D\left(\Xi \right)}$ is the posterior expectation of the deviance. It is calculated from the MCMC output by taking the sample mean of the simulated values of the deviance, $D\left(\hat{\Xi }\right)=-2\log p\left(y|\hat{\Xi }\right)$. That is defined as the deviance of the posterior estimation mean. Here $\hat{\Xi }$ denotes the posterior means of the parameters. The model with a smaller DIC has a better fit of the data.

## 4. Simulation Study

### 4.1. Simulation 1

This simulation study is conducted to evaluate the recovery performance of the slice-Gibbs sampling algorithm under different simulation conditions.

The following design conditions are considered: (a) testlet type: 20 dichotomous items in 2 or 4 testlets (*J* = 20, each testlet has 10 or 5 dichotomous items); (b) number of examinees, *N* = 500 and 1,000; and (c) testlet effect: the variances of the testlet random effect are 0.25 and 1.00. That is, $\sigma_{\eta_{ik}}^{2}=0.25$ or 1.00, where *i* = 1, …, *N*, *k* = 1, 2, or *k* = 1, 2, 3, 4. The true values of item discrimination parameters *a*_*j*_s are generated from a truncated normal distribution, that is, *a*_*j*_ ~ *N*(0, 1)I(0, +∞), and the item difficulty parameters *b*_*j*_s are generated from *N*(0, 1). Ability parameters θ_*i*_s for *N* = 500 or 1,000 examinees are drawn from a standard normal distribution. The testlets random effect parameters η_*ik*_s are also generated from a normal distribution. That is, $\eta_{ik}\sim N\left(0,\sigma_{\eta_{ik}}^{2}\right)$. Response data are simulated using the N2PLTM in Equation (1). The non-informative priors and hyper priors of parameters are considered as follows:

$$\begin{array}{l} a_{j}\sim N\left(0,100\right)\text{I}\left(0,+\infty \right),\;b_{j}\sim N\left(0,100\right),\;j=1,\ldots ,J,\\ \sigma_{a}^{2}\sim \text{IG}\left(0.001,0.001\right),\;\sigma_{b}^{2}\sim \text{IG}\left(0.001,0.001\right),\;\sigma_{\eta_{ik}}^{2}\\ \;\sim \text{IG}\left(0.001,0.001\right). \end{array}$$

The non-informative priors and hyper priors are often used in many educational measurement studies (e.g., van der Linden, 2007; Wang et al., 2018). In this paper, the prior specification will be uninformative enough for the data to dominate the priors, so that the influence of the priors on the results will be minimal.

#### 4.1.1. Convergence Diagnostic for Slice-Gibbs Algorithm

**Figure 1:**
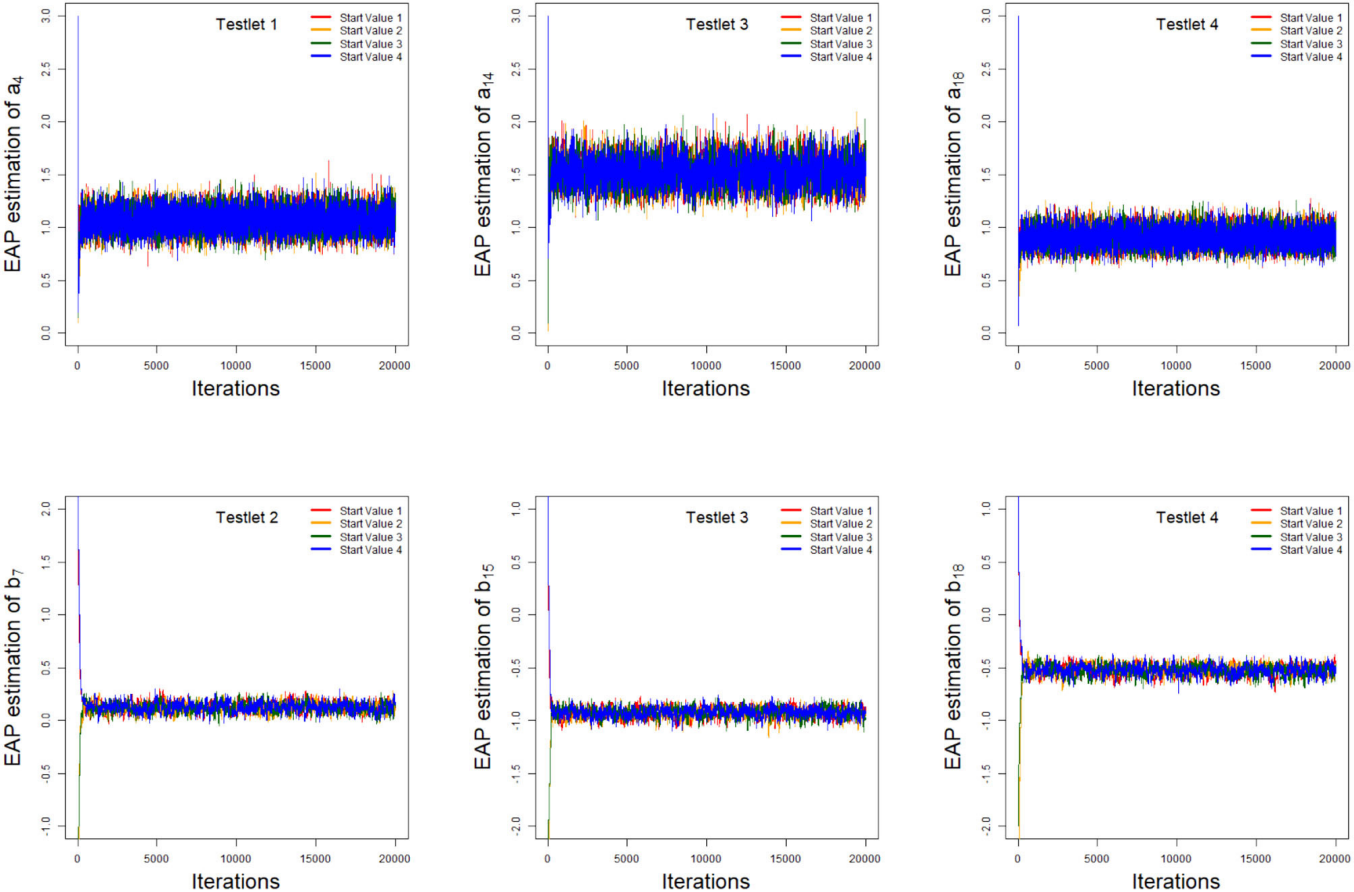
The trace plots of the arbitrarily selected item parameters.

**Figure 2:**
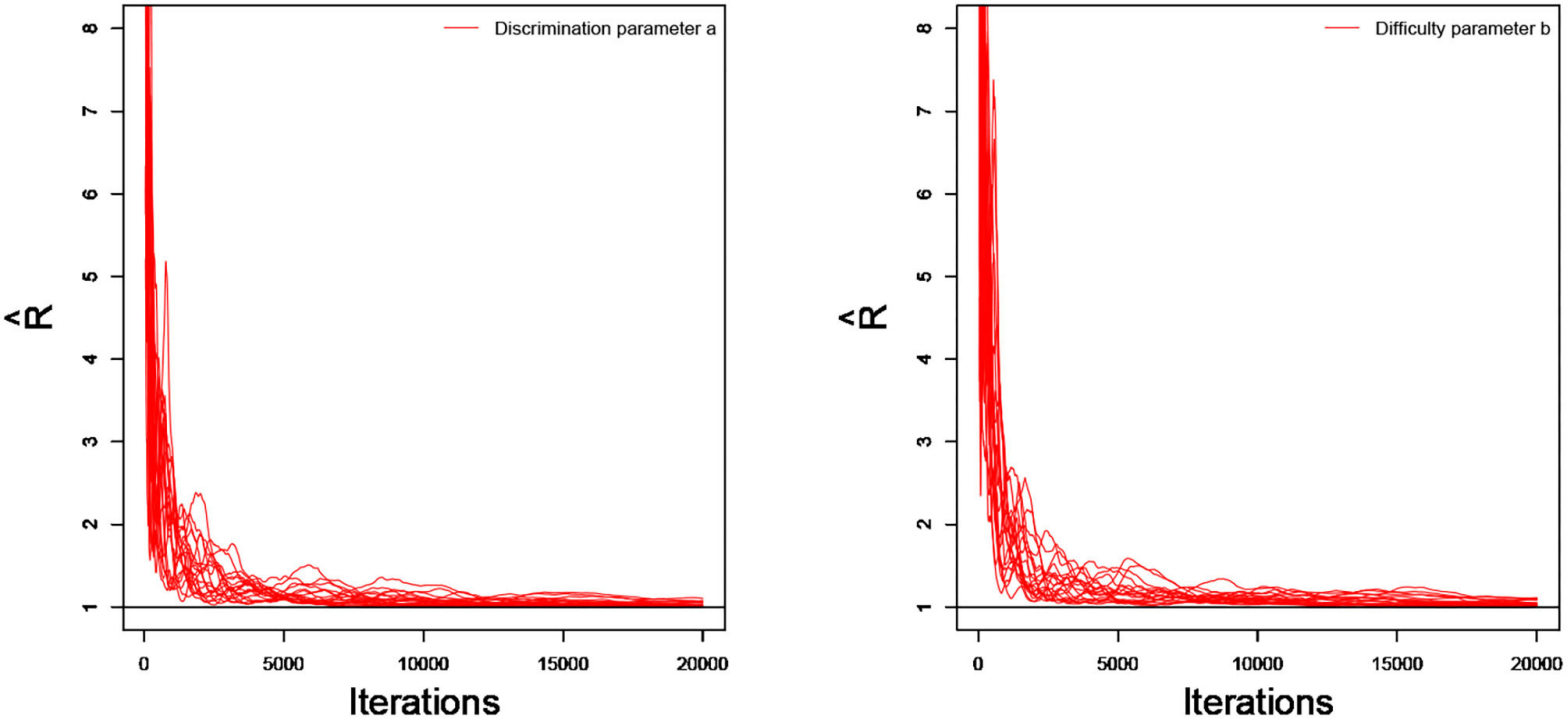
The trace plots of $\hat{R}$ for the simulation study 1.

As an illustration, we only consider the convergence in the case of 20 dichotomous items in 4 testlets, the number of individuals is 500, and the variance of the random testlet variables is 0.25. Two methods are used to check the convergence of our algorithm. One is the “eyeball” method to monitor the convergence by visually inspecting the history plots of the generated sequences (Zhang et al., 2007), and another method is to use the Gelman-Rubin method (Gelman and Rubin, 1992; Brooks and Gelman, 1998) to check the convergence of the parameters. Bayesian computation procedure is implemented by R software. The convergence of slice-Gibbs algorithm algorithm is checked by monitoring the trace plots of the parameters for consecutive sequences of 20,000 iterations. We set the first 10,000 iterations as the burn-in period. Four chains started at overdispersed starting values are run for each replication. The trace plots of item parameters randomly selected are shown in Figure 1. In addition, we find the potential scale reduction factor (PSRF; Brooks and Gelman, 1998) values of all parameters are <1.1, which ensures that all chains converge as expected. As an illustration, the PSRF values of all item parameters are shown in Figure 2. On a desktop computer [AMD EPYC 7542 32-Core Processor] with 2.90 GHz dual core processor and 1TB of RAM memory, the average convergence times for our new algorithm and the traditional Metropolis-Hastings algorithm based on 50 replications, are shown in Table 1.

**Table 1:** Convergence times for all 8 simulation conditions in simulation study 1.

**Sample size**	**Variance of**	**Time for convergence (Hours)**	
**×testlet type**	**testlet effect**	**Slice-Gibbs algorithm**	**MH algorithm**
500 × 2		0.2624	0.3182
500 × 4	0.25	0.4428	0.5864
1,000×2		0.3261	0.4639
1,000×4		0.6354	0.7882
500 × 2		0.2781	0.3325
500 × 4	1	0.6262	0.7691
1,000×2		0.4045	0.5952
1,000×4		0.8827	1.1201

*MH denotes the Metropolis-Hastings*.

#### 4.1.2. The Accuracy Evaluation of Parameter Estimation

The accuracy of the parameter estimates is measured by two evaluation methods, namely, Bias and mean squared error (MSE). The recovery results are based on the 50 replications in each simulation condition. The number of replication we choose is based on the previous research in educational psychological assessments. For example, Wang et al. (2013) proposed a semi-parametric approach, specifically, the Cox proportional hazards model with a latent speed covariate to analyze the response time data. In their simulation study, 10 replications (Page 15, section 4.1) are used for each simulation condition. Zhan et al. (2017) proposed joint modeling of attributes and response speed using item responses and response times simultaneously for cognitive diagnosis to provide more refined diagnostic feedback with collateral information in item response times. In their simulation study, they used 30 replications (Page 276) in each condition to reduce the random error. Lu et al. (2020) proposed a new mixture model for responses and response times with a hierarchical ability structure, which incorporates auxiliary information from other subtests and the correlation structure of the abilities to detect examinees' rapid guessing behavior. The recovery of the estimates was based on 20 replications (Page 14, section 5). Lu and Wang (2020) proposed to use an innovative item response time model as a cohesive missing data model to account for the two most common item nonresponses: not-reached items and omitted items. They considered 20 replications (Page 21) for each simulation condition. Therefore, based on the previous empirical conclusions, we adopt 50 replications in our simulation studies. If we consider a large number of replications, it is impossible to check the $\hat{R}$ values (potential scale reduction factor; PSRF, Brooks and Gelman, 1998) calculated from each simulated dataset (replication) to ensure the parameter convergence. It will be a huge work when the simulated conditions increase. Let ϑ be the parameter of interest. *S* = 50 data sets are generated. Also, let ${\hat{\vartheta }}^{\left(s\right)}$ denotes the posterior mean obtained from the *s*th simulated data set for *s* = 1, …, *S*.

The Bias for parameter ϑ is defined as

(17) $$\begin{array}{l} \text{Bias}\left(\vartheta \right)=\frac{1}{S}\overset{S}{\underset{s=1}{\sum }}\left({\hat{\vartheta }}^{\left(s\right)}-\vartheta \right), \end{array}$$

and the mean squared error (MSE) for parameter ϑ is defined as

(18) $$\begin{array}{l} \text{MSE}\left(\vartheta \right)=\frac{1}{S}\overset{S}{\underset{s=1}{\sum }}{\left({\hat{\vartheta }}^{\left(s\right)}-\vartheta \right)}^{2}. \end{array}$$

From Tables 2–4, the Bias is between −0.3267 and 0.2769 for the discrimination parameters, between –0.2259 and 0.2071 for the difficulty parameters, between −0.0132 and 0.0161 for the variance parameters of *****a*****, between −0.0219 and 0.1303 for the variance parameters of *****b*****, between −0.2932 and 0.0332 for the variance parameter of testlet effect η. the MSE is between 0.0000 and 0.1162 for the discrimination parameters, between 0.0000 and 0.0552 for the difficulty parameters, between 0.0002 and 0.0005 for the variance parameters of *****a*****, between 0.0002 and 0.0449 for the variance parameters of *****b*****, between 0.0000 and 0.1848 for the variance parameter of testlet effect η. In summary, the slice-Gibbs algorithm provides accurate estimates of the parameters in term of various numbers of examinees and items.

**Table 2:** Evaluating accuracy of the item parameter estimates based on different simulation conditions in the simulation study 1.

**The testlet effect with small variance** $\left(\sigma_{\eta_{k}}^{2}=0.25\right)$											
**Two testlets** (***k*** = 2)						**Four testlets** (***k*** = 4)					
		***N*** **= 500**		***N*** **= 1,000**				***N*** **= 500**		***N*** **= 1,000**	
**Testlets**	**Para**.	**Bias**	**MSE**	**Bias**	**MSE**	**Testlet**	**Para**.	**Bias**	**MSE**	**Bias**	**MSE**
	*a*_1_	0^*^	0^*^	0^*^	0^*^		*a*_1_	0^*^	0^*^	0^*^	0^*^
	*a*_2_	–0.0220	0.0122	–0.0596	0.0085		*a*_2_	–0.0901	0.0320	–0.0331	0.0036
	*a*_3_	0.1079	0.0259	0.0371	0.0053		*a*_3_	–0.0437	0.0172	–0.1163	0.0299
	*a*_4_	0.1293	0.0269	–0.0194	0.0100		*a*_4_	–0.0517	0.0116	–0.0217	0.0046
	*a*_5_	0.1430	0.0340	0.0201	0.0029	1	*a*_5_	0.0375	0.0080	0.0209	0.0030
	*a*_6_	0.0735	0.0211	0.0969	0.0236		*b*_1_	0 ^*^	0^*^	0^*^	0^*^
	*a*_7_	0.0296	0.0156	–0.0170	0.0058		*b*_2_	–0.0229	0.0012	–0.1338	0.0194
	*a*_8_	0.1060	0.0238	0.1418	0.0414		*b*_3_	–0.0100	0.0016	–0.0489	0.0027
	*a*_9_	0.0043	0.0119	–0.1767	0.0418		*b*_4_	0.0678	0.0059	0.0084	0.0013
1	*a*_10_	0.0044	0.0162	0.0155	0.0050		*b*_5_	–0.0338	0.0043	0.1382	0.0216
	*b*_1_	0^*^	0^*^	0^*^	0^*^		*a*_6_	0.0013	0.0055	-0.0099	0.0043
	*b*_2_	0.0784	0.0066	0.0595	0.0046		*a*_7_	–0.0321	0.0080	–0.0526	0.0121
	*b*_3_	–0.0999	0.0121	0.1838	0.0346		*a*_8_	–0.1421	0.0314	–0.0682	0.0195
	*b*_4_	–0.1049	0.0120	–0.0586	0.0043		*a*_9_	–0.1936	0.0484	–0.1320	0.02678
	*b*_5_	0.0572	0.0064	0.0648	0.0081	2	*a*_10_	–0.0459	0.0107	0.0698	0.0067
	*b*_6_	–0.0441	0.0030	–0.1098	0.0125		*b*_6_	0.0621	0.0088	–0.0551	0.0041
	*b*_7_	0.0233	0.0021	0.0139	0.0018		*b*_7_	–0.0227	0.0049	0.0557	0.0034
	*b*_8_	–0.0780	0.0078	–0.0950	0.0093		*b*_8_	0.0470	0.0042	0.0461	0.0024
	*b*_9_	0.0061	0.0007	–0.0145	0.0007		*b*_9_	–0.0519	0.0039	–0.1125	0.0129
	*b*_10_	0.0309	0.0018	0.0711	0.0073		*b*_10_	–0.0754	0.0105	0.1889	0.0382
	*a*_11_	–0.0930	0.0273	–0.0404	0.0079		*a*_11_	0.0132	0.0080	–0.0040	0.0064
	*a*_12_	–0.0566	0.0188	–0.0543	0.0109		*a*_12_	–0.0766	0.0253	–0.0105	0.0100
	*a*_13_	–0.0092	0.0112	0.0431	0.0266		*a*_13_	–0.0444	0.0111	0.0010	0.0077
	*a*_14_	0.0824	0.0223	–0.1066	0.0241		*a*_14_	–0.0838	0.0255	0.0694	0.0086
	*a*_15_	0.0670	0.0154	0.1983	0.0461	3	*a*_15_	–0.1910	0.0489	–0.0047	0.0060
	*a*_16_	0.0681	0.0201	–0.0650	0.0170		*b*_11_	–0.0746	0.0069	0.0572	0.0039
	*a*_17_	–0.0427	0.0116	0.2769	0.1023		*b*_12_	–0.0766	0.0064	0.0149	0.0006
	*a*_18_	0.0872	0.0183	0.1844	0.0403		*b*_13_	0.0983	0.0128	0.0247	0.0015
	*a*_19_	–0.0731	0.0164	–0.0246	0.0078		*b*_14_	–0.0384	0.0020	0.1116	0.0140
2	*a*_20_	0.0856	0.0149	–0.1472	0.0302		*b*_15_	0.1051	0.0121	–0.0203	0.0012
	*b*_11_	0.0018	0.0008	–0.1063	0.0120		*a*_16_	–0.1907	0.0522	–0.0602	0.0071
	*b*_12_	0.0254	0.0018	0.0042	0.0005		*a*_17_	0.0069	0.0057	–0.0596	0.0064
	*b*_13_	0.0404	0.0029	–0.1164	0.0137		*a*_18_	–0.0233	0.0084	–0.0467	0.0069
	*b*_14_	0.0545	0.0082	–0.0481	0.0032		*a*_19_	–0.1432	0.0368	–0.0512	0.0088
	*b*_15_	0.0118	0.0029	0.1903	0.0365	4	*a*_20_	–0.0780	0.0157	–0.1109	0.0276
	*b*_16_	–0.0168	0.0064	–0.0048	0.0006		*b*_16_	0.0351	0.0020	0.0784	0.0071
	*b*_17_	–0.0871	0.0084	0.1171	0.0139		*b*_17_	–0.1779	0.0372	–0.1403	0.0213
	*b*_18_	0.1374	0.0203	0.2071	0.0437		*b*_18_	0.0465	0.0052	–0.0353	0.0023
	*b*_19_	0.0175	0.0015	–0.0419	0.0030		*b*_19_	–0.0441	0.0029	–0.0976	0.0115
	*b*_20_	–0.0676	0.0091	–0.0582	0.0038		*b*_20_	0.0672	0.0057	0.0706	0.0054

Asterisks (*) indicates the constraints for model identifications. In fact, we need fix an item discrimination and difficulty parameters to one and zero due to model identifiability limitations. That is, a_1_ = 1, b_1_ = 0. In Bayesian estimation process, the Bias and MSE for the discrimination parameter a_1_ are blackened 0. Similarly, the Bias and MSE for the difficulty parameter b_1_ are also blackened 0.

**Table 3:** Evaluating accuracy of the item parameter estimates based on different simulation conditions in the simulation study 1.

**The testlet effect with large variance** $\left(\sigma_{\eta_{k}}^{2}=1.00\right)$											
**Two testlets** (***k*** = 2)						**Four testlets** (***k*** = 4)					
		***N*** **= 500**		***N*** **= 1,000**				***N*** **= 500**		***N*** **= 1,000**	
**Testlets**	**Para**.	**Bias**	**MSE**	**Bias**	**MSE**	**Testlet**	**Para**.	**Bias**	**MSE**	**Bias**	**MSE**
	*a*_1_	0^*^	0^*^	0^*^	0^*^		*a*_1_	0^*^	0^*^	0^*^	0^*^
	*a*_2_	0.1068	0.0532	–0.0423	0.0060		*a*_2_	–0.0206	0.0128	0.0762	0.0109
	*a*_3_	0.0399	0.0122	0.0120	0.0023		*a*_3_	0.0562	0.0201	–0.0674	0.0210
	*a*_4_	0.0665	0.0164	0.0684	0.0130		*a*_4_	0.0447	0.0137	0.0751	0.0137
	*a*_5_	0.0898	0.0185	0.0541	0.0096	1	*a*_5_	0.1790	0.0411	0.0915	0.0118
	*a*_6_	–0.0190	0.0139	0.1984	0.0573		*b*_1_	0 ^*^	0^*^	0^*^	0^*^
	*a*_7_	–0.0810	0.0258	0.0352	0.0063		*b*_2_	–0.0045	0.0008	–0.1139	0.0138
	*a*_8_	0.0113	0.0150	0.2475	0.0768		*b*_3_	0.0020	0.0011	–0.0247	0.0011
	*a*_9_	–0.1398	0.0369	–0.0888	0.0217		*b*_4_	0.0832	0.0079	0.0365	0.0024
1	*a*_10_	–0.1216	0.0358	0.0595	0.0061		*b*_5_	–0.0402	0.0040	0.1794	0.0338
	*b*_1_	0^*^	0^*^	0^*^	0^*^		*a*_6_	0.0562	0.0087	0.0709	0.0109
	*b*_2_	0.0777	0.0065	0.0795	0.0071		*a*_7_	0.0629	0.0155	–0.0408	0.0133
	*b*_3_	–0.0727	0.0086	0.1899	0.0367		*a*_8_	–0.1050	0.0237	–0.0317	0.0139
	*b*_4_	–0.0751	0.0063	–0.0479	0.0029		*a*_9_	–0.1127	0.0269	–0.0780	0.0225
	*b*_5_	0.0535	0.0067	0.1047	0.0136	2	*a*_10_	0.0696	0.0128	0.1520	0.0259
	*b*_6_	–0.0293	0.0017	–0.1021	0.0107		*b*_6_	0.1359	0.0237	–0.0591	0.0045
	*b*_7_	0.0236	0.0020	0.0503	0.0042		*b*_7_	0.0162	0.0028	0.0435	0.0022
	*b*_8_	–0.0498	0.0039	–0.0962	0.0094		*b*_8_	0.0954	0.0110	0.0344	0.0016
	*b*_9_	0.0044	0.0009	0.0047	0.0004		*b*_9_	–0.0048	0.0007	–0.0918	0.0086
	*b*_10_	0.0291	0.0020	0.1053	0.0130		*b*_10_	–0.0405	0.0045	0.1919	0.0398
	*a*_11_	–0.1291	0.0416	–0.0248	0.0064		*a*_11_	0.2072	0.0521	0.1561	0.0371
	*a*_12_	–0.0855	0.0248	–0.0099	0.0092		*a*_12_	0.0261	0.0241	0.1212	0.0288
	*a*_13_	–0.0509	0.0204	0.0114	0.0120		*a*_13_	0.0070	0.0086	0.1183	0.0262
	*a*_14_	0.0745	0.0147	–0.0630	0.0124		*a*_14_	0.0525	0.0187	0.2235	0.0569
	*a*_15_	0.0388	0.0098	0.2199	0.0528	3	*a*_15_	–0.3267	0.1162	0.1419	0.0311
	*a*_16_	0.0719	0.0139	–0.0337	0.0127		*b*_11_	–0.1127	0.0143	0.0245	0.0011
	*a*_17_	0.0412	0.0331	0.2466	0.0734		*b*_12_	–0.0932	0.0093	–0.0246	0.0011
	*a*_18_	0.1039	0.0226	0.2060	0.0462		*b*_13_	0.1460	0.0230	–0.0192	0.0018
	*a*_19_	–0.1304	0.0333	0.0110	0.0102		*b*_14_	–0.0334	0.0020	0.0751	0.0066
2	*a*_20_	0.0585	0.0105	–0.1228	0.0251		*b*_15_	0.1157	0.0152	–0.0727	0.0059
	*b*_11_	–0.0149	0.0015	–0.1035	0.0117		*a*_16_	–0.1712	0.0499	0.0534	0.0091
	*b*_12_	0.0055	0.0014	–0.0064	0.0005		*a*_17_	0.1437	0.0265	0.0320	0.0052
	*b*_13_	0.0277	0.0024	–0.0992	0.0100		*a*_18_	0.0859	0.0176	0.0934	0.0141
	*b*_14_	0.0286	0.0064	–0.0508	0.0032		*a*_19_	–0.1100	0.0306	0.0515	0.0080
	*b*_15_	–0.0027	0.0033	0.1773	0.03176	4	*a*_20_	–0.0396	0.0180	–0.1562	0.0377
	*b*_16_	–0.0326	0.0062	–0.0109	0.0006		*b*_16_	0.0542	0.0037	0.1187	0.0151
	*b*_17_	–0.0887	0.0087	0.1086	0.0121		*b*_17_	–0.2259	0.0552	–0.1822	0.0344
	*b*_18_	0.1242	0.0168	0.1821	0.0336		*b*_18_	0.0843	0.0099	–0.0397	0.0023
	*b*_19_	0.0057	0.0015	–0.0529	0.0040		*b*_19_	–0.0275	0.0020	–0.1136	0.0137
	*b*_20_	–0.0580	0.0073	–0.0641	0.0046		*b*_20_	0.1055	0.0123	0.0684	0.0050

Asterisks (*) indicates the constraints for model identifications. In fact, we need fix an item discrimination and difficulty parameters to one and zero due to model identifiability limitations. That is, a_1_ = 1, b_1_ = 0. In Bayesian estimation process, the Bias and MSE for the discrimination parameter a_1_ are blackened 0. Similarly, the Bias and MSE for the difficulty parameter b_1_ are also blackened 0.

**Table 4:** Evaluating accuracy of the variance parameter estimates.

**The testlet effect with small variance** $\left(\sigma_{\eta_{k}}^{2}=0.25\right)$									
**Two testlets** (***k*** = 2)					**Four Testlets** (***k*** = 4)				
	***N*** **= 500**		***N*** **= 1,000**			***N*** **= 500**		***N*** **= 1,000**	
**Para**.	**Bias**	**MSE**	**Bias**	**MSE**	**Para**.	**Bias**	**MSE**	**Bias**	**MSE**
$\sigma_{a}^{2}$	0.0161	0.0005	0.0080	0.0003	$\sigma_{a}^{2}$	0.0079	0.0002	–0.0092	0.0002
$\sigma_{b}^{2}$	–0.0219	0.0005	0.2119	0.0449	$\sigma_{b}^{2}$	0.0572	0.0033	0.1303	0.0170
$\sigma_{\eta_{1}}^{2}$	0.0283	0.0008	0.0209	0.0004	$\sigma_{\eta_{1}}^{2}$	–0.0051	0.0000	–0.0029	0.0000
$\sigma_{\eta_{2}}^{2}$	0.0234	0.0005	0.0332	0.0011	$\sigma_{\eta_{2}}^{2}$	–0.0021	0.0000	–0.0024	0.0000
					$\sigma_{\eta_{3}}^{2}$	–0.0102	0.0001	–0.0054	0.0000
					$\sigma_{\eta_{4}}^{2}$	–0.0059	0.0000	–0.0092	0.0000
**The testlet effect with large variance** $\left(\sigma_{\eta_{k}}^{2}=1.00\right)$									
**Two testlets** (*k* = 2)					**Four testlets** (*k* = 4)				
	***N*** **= 500**		***N*** **= 1,000**			***N*** **= 500**		***N*** **= 1,000**	
**Para**.	**Bias**	**MSE**	**Bias**	**MSE**	**Para**.	**Bias**	**MSE**	**Bias**	**MSE**
$\sigma_{a}^{2}$	0.0106	0.0005	0.0094	0.0002	$\sigma_{a}^{2}$	0.0053	0.0002	–0.0132	0.0003
$\sigma_{b}^{2}$	–0.0135	0.0002	0.2181	0.0475	$\sigma_{b}^{2}$	0.0398	0.0016	0.1336	0.0178
$\sigma_{\eta_{1}}^{2}$	–0.1955	0.0382	–0.1953	0.0382	$\sigma_{\eta_{1}}^{2}$	–0.2333	0.1112	–0.2104	0.0964
$\sigma_{\eta_{2}}^{2}$	–0.2254	0.0509	–0.2014	0.0405	$\sigma_{\eta_{2}}^{2}$	–0.2932	0.0863	–0.2241	0.1051
					$\sigma_{\eta_{3}}^{2}$	–0.2194	0.1760	–0.2298	0.1848
					$\sigma_{\eta_{4}}^{2}$	–0.2024	0.1622	–0.2177	0.1745

### 4.2. Simulation 2

This simulation study is designed to show that the slice-Gibbs sampling algorithm is sufficiently flexible to recover various prior distributions of the item parameters and address the sensitivity of our slice-Gibbs algorithm with different priors.

Response pattern with 500 examinees and 4 testlets (5 items per testlet) is generated by N2PLTM as given by Equation (1). The true values of item parameters and ability parameters are generated same as in simulation 1. The true value of the testlet effect variance is set equal to 0.25. The specified types of item parameter priors are given by the following:

**Type I**: Informative priors, *a*_*j*_ ~ *N*(0, 1)I(0, +∞) and *b*_*j*_ ~ *N*(0, 1);
**Type II**: Noninformative priors, *a*_*j*_ ~ *N*(0, 100)I(0, +∞) and *b*_*j*_ ~ *N*(0, 100);
**Type III**: Noninformative priors, *a*_*j*_ ~ Uniform(0, 100) and *b*_*j*_ ~ Uniform(0, 100).

Prior specifications for the other parameters are identical to the simulation study 1. To implement the MCMC sampling algorithm, chains of length 20,000 with an initial burn-in period 10,000 are chosen, and the PSRF values of all parameters are <1.1. Based on 25 replications, the average times for all parameters to converge in Type I, Type II and Type III are 0.4597, 0.4428, and 0.4506 h, respectively.

The average Bias and MSE for item parameters based on 50 replication are shown in Table 5. We find that the average Bias and MSE for item parameters are relatively unchanged under the three different prior distributions. The slice-Gibbs sampling algorithm allows for informative (Type I) or non-informative (Type II, Type III) priors of the item parameters and is not sensitive to the specification of priors. Moreover, a wider range of prior distributions is also appealing.

**Table 5:** Average Bias and MSE for the item parameter estimates using three prior distributions in the simulation study 2.

	**Type I**		**Type II**		**Type III**	
**Parameter**	**Bias**	**MSE**	**Bias**	**MSE**	**Bias**	**MSE**
Discrimination ***a***	–0.0757	0.0250	–0.0641	0.0245	–0.0695	0.0260
Difficulty ***b***	–0.0039	0.0064	–0.0038	0.0064	–0.0038	0.0065

### 4.3. Simulation 3

In this simulation study, we will investigate the power of the model assessment methods. Namely, whether the Bayesian model comparison criteria based on the MCMC output could identify the true model from which the data are generated. The simulation design is as follows.

A data set with 500 examinees from standard normal distribution and four testlets (five items per testlet) is generated from the N2PLTM model. For the true values of parameters, the discrimination parameters *a*_*j*_*s* are generated from the truncated normal distribution, that is, *a*_*j*_ ~ *N*(0, 1)I(0, +∞). The difficulty parameters *b*_*j*_*s* are generated from normal distribution, that is, *b*_*j*_ ~ *N*(0, 1). The independent-items model as Model 1 is used to model assessment in which the random effects are set to zero. Model 1 is known as two parameter logistic model (2PLM; Birnbaum, 1957). In addition, the testlets random effect parameters η_*ik*_s are generated from a normal distribution. That is, η_*ik*_ ~ *N*(0, 0.25), *k* = 1, 2, 3, 4. Model 2 is the traditional two parameter logistic testlet model (T2PLTM; Bradlow et al., 1999), which is give by

(19) $$\begin{array}{l} p_{ij}=p\left(y_{ij}=1|\theta_{i},a_{j},b_{j},\eta_{id\left(j\right)}\right)=\frac{\exp\left[a_{j}\left(\theta_{i}-b_{j}+\eta_{id\left(j\right)}\right)\right]}{1+\exp\left[a_{j}\left(\theta_{i}-b_{j}+\eta_{id\left(j\right)}\right)\right]}. \end{array}$$

Model 3 is the N2PLTM in Equation (1). The parameter priors are identical to the simulation study 1. The parameters are estimated based on 20,000 iterations after a 10,000 burn-in period, and the PSRF values of all parameters are <1.1. Two Bayesian model assessment methods are used to model fitting. That is, DIC and log-PsBF. The results of Bayesian model assessment based on 50 replications are shown in Table 6.

**Table 6:** The results of Bayesian model assessment in the simulation 3.

	**Fitted model**			**Model 1 (2PL)**	**Model 2 (T2PLT)**	**Model 3 (N2PLT)**
True	Model 3		*Q*_1_	11380.77	11124.27	11065.03
model	(N2PLT)	DIC	Median	11412.16	11153.87	11098.49
			*Q*_3_	11488.77	11226.28	11159.71
			IQR	107.99	102.01	94.67
			*Q*_1_	−5-903.97	–5658.31	–5634.16
		log-PsBF	Median	–5870.39	–5620.26	–5595.36
			*Q*_3_	–5856.31	–5604.20	–5590.11
			IQR	47.65	54.11	44.05

From Table 6, we find that when the Model 3 (N2PLTM model) is the true model, the Model 3 is chosen as the best-fitting model according to the results of the DIC and log-PsBF, which is what we expect to see. The medians of DIC and log-PsBF are respectively 11098.49 and −5595.36. The Model 2 (T2PLTM model) is the second best fitting model, which is attributed to the fact that the Model 2 with testlet random effect as well as the Model 3 also can capture the dependency structure between items. The differences between Model 3 and Model 2 in the median of DIC and log-PsBF are −55.38 and 24.9, respectively. However, compared the T2PLTM model, the N2PLTM model with the testlet discrimination parameter α is more flexible and the fitting is more sufficient. The Model 1 (2PL model) is worst-fitting model. The medians of DIC and log-PsBF are respectively 11412.16 and −5870.39. The differences between Model 3 model and Model 1 in the median of DIC and log-PsBF are −313.67 and 275.03, respectively. This is because the Model 1 do not consider the complicated and interrelated sets of items, thus it can not improve the model fitting for the testlet item response data. In summary, the Bayesian assessment criteria is effective for identifying the true models and it can be used in the subsequent empirical example analysis.

## 5. Empirical Example

To illustrate the applicability of the testlet IRT modeling method to large-scale test assessments, we consider a data set of students' English reading comprehension test for Maryland university (Tao et al., 2013). A total of 1,289 students take part in the test and answer 28 items. The 28 items consist of 4 testlets. Testlet 1 is formed by Items 1 to 8, that is, *d*(1) = ⋯ = *d*(8) = 1; Testlet 2 by Items 9 to 15, that is, *d*(9) = ⋯ = *d*(15) = 2; Testlet 3 by Items 16 to 23, that is, *d*(16) = ⋯ = *d*(23) = 3; and Testlet 4 by Items 24–28, that is, *d*(24) = ⋯ = *d*(28) = 4. The following prior distributions are used to analyze the data. That is,

$$\begin{array}{l} a_{j}\sim N\left(0,100\right)\text{I}\left(0,+\infty \right),\;b_{j}\sim N\left(0,100\right),\;j=1,\ldots ,28,\\ \theta_{i}\sim N\left(0,1\right),\;\eta_{id\left(j\right)}\sim N\left(0,1\right),\;i=1,\ldots ,1289,\;j=1,\ldots ,28. \end{array}$$

We consider three models to fit the real data. The three models are 2PLM, T2PLTM and N2PLTM, respectively. The slice-Gibbs algorithm is applied to estimate the parameters of the three models. The slice-Gibbs sampling is iterated 20,000 iterations, with a burn-in period of 10,000 iterations. The convergence of the chains is checked by PSRF, which are <1.1. The item parameters of the N2PLTM are estimated and the item parameter estimators and the corresponding standard deviations are provided in Table 7. In the Bayesian frame work, the 95% highest posterior density intervals (HPDI) are calculated as confidence regions for the item parameters and are given in the columns labeled HPDI_*a*_ and HPDI_*b*_ in Table 8.

**Table 7:** The results of Bayesian model assessment in the real data.

**Model**	**DIC**	**log-PsBF**
2PLM	44179.93	–22021.39
T2PLTM	40796.35	–20794.23
N2PLTM	**40632.52**	**–20708.47**

*The meaning of the bold values is the best fitting model*.

**Table 8:** The estimation results of item parameter for the real data.

**Testlets**	**Para**.		**EAP**		**SD**		**HPDI**	
	**a**	**b**	$\hat{a}$	$\hat{b}$	**SD_***a***_**	**SD_***b***_**	**HPDI_***a***_**	**HPDI_*b*_**
1	*a*_1_	*b*_1_	1.0000	0.0000	0.0000	0.0000	[1.0000, 1.0000]	[0.0000, 0.0000]
1	*a*_2_	*b*_2_	1.6319	0.2606	0.0116	0.0001	[1.4281, 1.8411]	[0.2308, 0.2845]
1	*a*_3_	*b*_3_	0.7215	0.7808	0.0053	0.0017	[0.5837, 0.8673]	[0.6971, 0.8575]
1	*a*_4_	*b*_4_	0.6302	-0.2913	0.0033	0.0015	[0.5278, 0.7525]	[−0.3747, −0.2197]
1	*a*_5_	*b*_5_	0.8039	0.6052	0.0062	0.0007	[0.6385, 0.9471]	[0.5509, 0.6577]
1	*a*_6_	*b*_6_	0.7998	0.6283	0.0046	0.0010	[0.6667, 0.9380]	[0.5528, 0.6832]
1	*a*_7_	*b*_7_	1.1367	0.2697	0.0066	0.0004	[0.9717, 1.2945]	[0.2261, 0.3114]
1	*a*_8_	*b*_8_	1.1849	–0.0253	0.0053	0.0006	[1.0291, 1.3164]	[−0.0760, 0.0236]
2	*a*_9_	*b*_9_	0.8047	–0.7197	0.0018	0.0013	[0.7168, 0.8845]	[−0.7981, −0.6511]
2	*a*_10_	*b*_10_	0.6128	–0.7850	0.0016	0.0030	[0.5314, 0.6864]	[−0.8908, −0.6853]
2	*a*_11_	*b*_11_	1.6674	–0.0463	0.0069	0.0002	[1.5081, 1.8327]	[−0.0772, −0.0140]
2	*a*_12_	*b*_12_	1.0907	–0.2133	0.0076	0.0024	[0.9463, 1.2035]	[−0.3290, −0.1994]
2	*a*_13_	*b*_13_	1.7084	0.0546	0.0099	0.0001	[1.5124, 1.9014]	[0.0292, 0.0800]
2	*a*_14_	*b*_14_	1.0951	–0.0775	0.0047	0.0007	[0.9635, 1.2267]	[−0.1271, −0.0213]
2	*a*_15_	*b*_15_	0.9024	–0.1817	0.0042	0.0013	[0.7719, 1.0226]	[−0.2476, −0.1093]
3	*a*_16_	*b*_16_	0.6347	0.5639	0.0057	0.0011	[0.4895, 0.7859]	[0.4997, 0.6370]
3	*a*_17_	*b*_17_	0.7751	0.1933	0.0058	0.0011	[0.6331, 0.9295]	[0.1275, 0.2588]
3	*a*_18_	*b*_18_	1.5116	–0.6624	0.0045	0.0004	[1.3786, 1.6420]	[−0.7092, −0.6226]
3	*a*_19_	*b*_19_	0.4526	0.5646	0.0040	0.0023	[0.3234, 0.5688]	[0.4703, 0.6521]
3	*a*_20_	*b*_20_	0.6325	0.7146	0.0054	0.0017	[0.4886, 0.7769]	[0.6321, 0.7972]
3	*a*_21_	*b*_21_	0.9391	–0.7392	0.0024	0.0011	[0.8374, 1.0301]	[−0.8025, −0.6775]
3	*a*_22_	*b*_22_	1.0175	–0.2715	0.0036	0.0008	[0.8983, 1.1347]	[−0.3266, −0.2105]
3	*a*_23_	*b*_23_	1.0722	–0.3727	0.0037	0.0009	[0.9526, 1.1831]	[−0.4389, −0.3178]
4	*a*_24_	*b*_24_	2.0055	–0.0069	0.0116	0.0002	[1.7917, 2.2080]	[−0.0349, 0.0216]
4	*a*_25_	*b*_25_	0.7821	0.4765	0.0052	0.0011	[0.6391, 0.9178]	[0.4068, 0.5391]
4	*a*_26_	*b*_26_	1.5236	0.2656	0.0103	0.0002	[1.3277, 1.7270]	[0.2388, 0.2969]
4	*a*_27_	*b*_27_	1.1934	0.3662	0.0084	0.0003	[1.0189, 1.3794]	[0.3316, 0.4050]
4	*a*_28_	*b*_28_	0.6847	–0.1442	0.0045	0.0016	[0.5563, 0.8153]	[−0.2222, −0.0667]

*Para. denotes the interest parameters. EAP denotes the expected a priori estimation. SD denotes the standard deviation. HPDI denotes the 95% highest posterior density intervals*.

Based on the results of Bayesian model selection form Table 7, we find that the N2PLTM is the best fitting model compared to the other two models. The DIC and log-PsBF are respectively 40632.52 and −20708.47. The second best fitting model is T2PLTM. The differences between N2PLTM and T2PLTM in the DIC and log-PsBF are −163.83 and 85.76, respectively. The 2PL model is worst-fitting model. The DIC and log-PsBF are respectively 44179.93 and –22021.39.

From Table 8, we find that for each testlet, the four items with highest discrimination are 2, 13, 18, and item 24, respectively. The expected a posteriori (EAP) estimations for the four item discrimination parameters are 1.6319, 1.7084, 1.5116, and 2.0055. The four most difficult items in each testlet are 3, 13, 20, and item 25 in turn. The EAP estimations for the four item difficulty parameters are 0.7808, 0.0546, 0.7146, and 0.4765. Compared to the items in the other three testlets, the items in the testlet 2 are relatively easy because the EAP estimates of the difficulty parameters (*b*_9_, *b*_10_, *b*_11_, *b*_12_, *b*_14_, and *b*_15_) are <0. In addition, the SD is between 0.0000 and 0.0116 for the discrimination parameters, between 0.0000 and 0.0030 for the difficulty parameters.

## 6. Concluding Remarks

To explore the relations between items with dependent structure, this current study proposes a N2PLTM and presents a effective Bayesian sampling algorithm. More specifically, an improved Gibbs sampling algorithm based on auxiliary variables is developed for estimating N2PLTM. The slice-Gibbs sampling algorithm overcomes the traditional Gibbs sampling algorithm's dependence on the conjugate prior for complex IRT model, and avoids some shortcomings of the Metropolis algorithm (such as sensitivity to step size, severe dependency on the candidate function or tuning parameter). Based on different simulation conditions, we find that the slice-Gibbs sampling algorithm can provide accurate parameter estimates in the sense of having small Bias and MSE values. In addition, the average Bias and MSE for item parameters are relatively unchanged under the three different prior distributions. The slice-Gibbs sampling algorithm allows for informative or non-informative priors of the item parameters and is not sensitive to the specification of priors. In summary, the algorithm is effective and can be used to analyze the empirical example.

However, the computational burden of the slice-Gibbs sampling algorithm becomes intensive especially when a large number of examinees or the items is considered, or a large number of the MCMC sample size is used. Therefore, it is desirable to develop a standing-alone R package associated with C++ or Fortran software for more extensive large-scale assessment program.

In addition, the new algorithm based on auxiliary variables can be extended to estimate some more complex item response and response time models, e.g., graded response model, Weibull response time model and so on.

## Data Availability Statement

The datasets analyzed in this manuscript are not publicly available. Requests to access the datasets should be directed to Bao Xu, xubao97@163.com.

## Author Contributions

JL and JZ completed the writing of the article, original thoughts, and provided key technical support. JL and ZZ provided key technical support. BX provided the data. JT and JL completed the article revisions. All authors contributed to the article and approved the submitted version.

## Conflict of Interest

The authors declare that the research was conducted in the absence of any commercial or financial relationships that could be construed as a potential conflict of interest.

## Publisher's Note

All claims expressed in this article are solely those of the authors and do not necessarily represent those of their affiliated organizations, or those of the publisher, the editors and the reviewers. Any product that may be evaluated in this article, or claim that may be made by its manufacturer, is not guaranteed or endorsed by the publisher.
